# An Update of Orthopoxvirus Molecular Evolution

**DOI:** 10.3390/v14020388

**Published:** 2022-02-14

**Authors:** Igor V. Babkin, Irina N. Babkina, Nina V. Tikunova

**Affiliations:** 1 Laboratory of Molecular Microbiology, Institute of Chemical Biology and Fundamental Medicine, 630090 Novosibirsk, Russia; 2 JSC VECTOR-BEST, 630559 Novosibirsk, Russia; irinanik_babkina@mail.ru

**Keywords:** variola virus, *orthopoxvirus*, evolution, origin

## Abstract

Although variola virus (VARV) has been eradicated through widespread vaccination, other *orthopoxviruses* pathogenic for humans circulate in nature. Recently, new *orthopoxviruses*, including some able to infect humans, have been found and their complete genomes have been sequenced. Questions about the *orthopoxvirus* mutation rate and the emergence of new threats to humankind as a result of the evolution of circulating *orthopoxviruses* remain open. Based on contemporary data on ancient VARV DNA and DNA of new *orthopoxvirus* species, an analysis of the molecular evolution of *orthopoxviruses* was carried out and the timescale of their emergence was estimated. It was calculated that the *orthopoxviruses* of the Old and New Worlds separated approximately 40,000 years ago; the recently discovered Akhmeta virus and Alaskapox virus separated from other *orthopoxviruses* approximately 10,000–20,000 years ago; the rest of modern orthopoxvirus species originated from 1700 to 6000 years ago, with the exception of VARV, which emerged in approximately 300 AD. Later, there was a separation of genetic variants of some *orthopoxvirus* species, so the monkeypox virus West African subtype originated approximately 600 years ago, and the VARV minor alastrim subtype emerged approximately 300 years ago.

## 1. Introduction

*Orthopoxvirus* is the most notorious genus of the *Poxviridae* family, which comprises complex DNA viruses. Several viruses of the genus represent a potential threat to humans. The most dangerous member of this genus has been variola virus (VARV), which can cause smallpox. According to various estimations, smallpox killed 300–500 million people in the 20th century [1,2]. By 1977, this virus had been eradicated everywhere as a result of a wide-scale vaccination campaign with live vaccinia virus (VACV) [1,3]. However, the threat of VARV use for bioterrorism purposes remains. Published studies describe the possibility of developing an artificially modified VARV capable of overcoming vaccine protection [4]. Monkeypox virus (MPXV) is another orthopoxvirus that can cause outbreaks of smallpox-like zoonotic disease. In recent decades, outbreaks of monkeypox have been recorded in the world [5,6], and there is evidence that the danger of MPXV to humans increases in the process of natural evolution [5]. Orthopoxviruses can be used as a biological weapon as, after smallpox eradication, large-scale vaccination with VACV was ended and most people under 45 years old have not been protected against *orthopoxvirus* infections. Besides VARV and MPXV, cowpox virus (CPXV) and VACV can infect humans; however, generalized infection can appear in individuals with impaired immunity. CPXV can infect many mammals, and various genetic variants of CPXV circulate in nature. Researchers believe that the ancestor of CPXV, with a wide range of susceptible hosts, is the progenitor of most of the currently studied specialized Old World *orthopoxviruses*. The main vectors of CPXV are numerous rodent species of a variety of habitats. This is a condition for the molecular evolution and diversity of CPXV and creates the possibility of the emergence of new *orthopoxviruses* pathogenic for humans and other mammals [7,8].

The range of studied *orthopoxviruses* is expanding constantly. In addition to the aforementioned human pathogenic *orthopoxviruses*, Abatino virus, Akhmeta virus, and Alaskapox virus have recently been discovered in Italy, Georgia, and the United States, respectively [9,10,11]. The last virus was probably imported from the Old World region [10]. Akhmeta virus and Alaskapox virus form separate branches from the rest of the Old World *orthopoxviruses* on the species phylogram of this genus, whereas Abatino clusters with ectromelia virus (ECTV). Progress in molecular methods has made it possible to study the genomic sequences of ancient VARV [2,12,13,14,15,16]. All these expanded data allow us to re-evaluate the molecular evolution of *orthopoxviruses* and more reliably determine their time of origin and the rate of genetic variation.

A number of studies have been devoted to assessing the evolutionary rate of *orthopoxviruses* and VARV in particular. In the previous studies, the rate of evolution has been calculated based on the central conservative region of the genome. The rate of mutation accumulation in the genomes of *orthopoxviruses* has been estimated to be approximately (4–6) × 10^−6^ substitutions/site/year [17]. The first estimate for VARV was approximately (2–4) × 10^−6^ substitutions/site/year and the time of VARV occurrence was calculated as 3000–4000 years ago [18,19,20]. With the expansion of the database of complete VARV genomes, studies have appeared based on the isolation times of various VARV strains. Firth et al. [21] estimated the rate of evolution of genome-wide VARV sequences based on the dates of virus strains isolation at 1 × 10^−5^ substitutions/site/year. However, all analyzed VARV strains were isolated in the 20th century.

Recently, ancient VARV DNA has been isolated from samples of a Lithuanian child mummy dating between 1643 and 1665 [12], from two specimens from the Czech National Museum dating about 160 years ago [14], and from four northern European individuals buried in the Viking Age, ~600–1000 AD [2], and their genome-wide sequencing has been carried out. In each case, this allowed for a more accurate dating of the evolutionary history of VARV [2,12,14]; the rates of the VARV genome evolution were calculated to be (7–9) × 10^−6^ substitutions/site/year [12] and (3.7–6.5) × 10^−6^ [2]. Later, dating for the Czech specimens was revised and they were estimated to be 1937 and 1933 under a relaxed clock [13,16]. The corrected rate of evolution of VARV complete genome sequences, with the exception of strains from the Czech Republic, was (7.0–10.0) × 10^−^^6^ substitutions/site/year [16].

In all these studies, the evolutionary history was reconstructed based on the complete VARV genomes, which can introduce distortions in chronograms due to the different contribution of the terminal variable regions of the VARV genome to the reconstruction of evolution and the high probability of recombination events in these regions of the genome [22,23]. Taking into consideration these data, the discovery of previously unknown *orthopoxviruses*, and new data on ancient viral DNA, we re-evaluated the timing of the VARV and other *orthopoxviruses* and their molecular evolution.

## 2. Materials and Methods

### 2.1. Retrieval of Genome Sequences and Alignment

Nucleotide sequences of *orthopoxviruses* were extracted from the NCBI database (http://www.ncbi.nih.gov, accessed on 12 September 2021). The *orthopoxvirus* strains analyzed in this work are listed in Table 1. Strains with a long passage history, such as CPXV Brighton [24] and all VACV strains, were excluded from this study. The exception was the VACV MNR-76 strain isolated from the horse and considered related to the VACV progenitor [25]. In addition, potentially recombinant strains such as CPXV Germany 1998_2 [7] were excluded. All *orthopoxvirus* strains included in the analysis had reliable collection dates.

The sequences of the highly conserved central genome region flanked by the open reading frames (ORFs) *F4L* and *A24R* (according to the nomenclature of the VACV strain Copenhagen) were isolated from genome sequences of *orthopoxviruses* (Table 1) and aligned using MAFFT [14].

### 2.2. Phylogenetic Tree Construction and Evolutionary Analyses

A maximum likelihood phylogenetic tree was constructed by using the IQ-TREE 2.0 software (http://iqtree.cibiv.univie.ac.at/, accessed on 10 November 2021) [26] in association with ModelFinder. The best-fit model TVM+F+I+G4 was chosen according to the Bayesian information criterion (BIC). The support for the inferred relationships was evaluated by applying the Shimodaira–Hasegawa-like approximate likelihood ratio test (SH-aLRT) [27] and bootstrap analysis with 500 bootstrap replicates. The final phylogeny was visualized in FigTree v1.4.3.

Molecular dating was performed by using the Bayesian Markov chain Monte Carlo (MCMC) inference method with BEAST 2 (Bayesian evolutionary analysis by sampling trees) software [28]. BEAST 2 was run using a log-normal relaxed clock and coalescent Bayesian skyline population prior. Sufficient MCMC chains were run (1 billion steps) to ensure convergence, with the initial 10% of the MCMC chains discarded as burn-in. Proper mixing of the Markov chain was assessed by calculating the effective sampling size (ESS) with Tracer software (BEAST 2 package). The maximum clade credibility tree was generated under a HKY substitution model with unequal base frequencies, invariant sites, and gamma-distributed rate heterogeneity among sites. This model most reliably describes the variation in coding genomic sequences [29]. Demographic plots for each analysis were visualized in Tracer v 1.7 [30]. The degree of the clock-like structure in the data was assessed based on a regression of root-to-tip genetic distances against a year of sampling using the TempEst program [31].

## 3. Results

### 3.1. Phylogenetic Analysis of Orthopoxviruses

To study the molecular evolution of *orthopoxviruses*, various species and strains of *orthopoxviruses* were selected, including currently known strains of different genotypes of *orthopoxviruses* and all unique VARV strains (Table 1). Notably, strains with a long passage history and potentially recombinant strains were excluded. Molecular evolution analysis was performed based on the central conserved region of the *orthopoxvirus* genome (approximately 102 kb).

**Figure 1:**
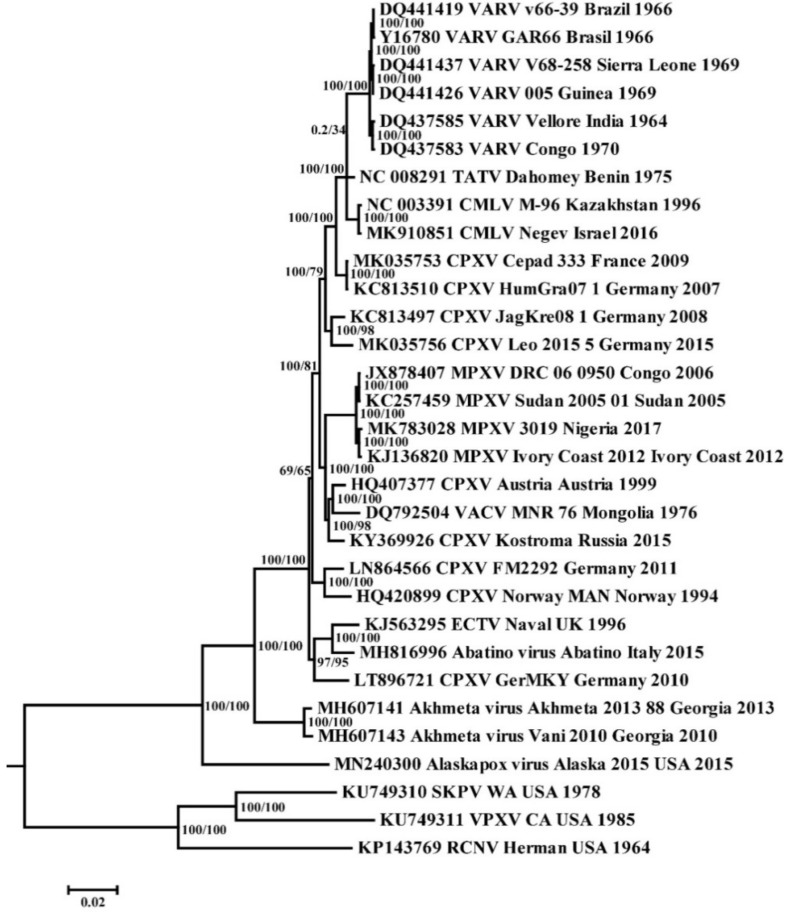
Phylogenetic tree for the highly conserved central genome region of the *orthopoxviruses* was generated using the maximum-likelihood method. Numbers above branches are SH-aLRT support (%)/bootstrap support (%). Divergence (substitutions per site) scales are given at the bottom. The strains are designated as in Table 1.

Phylogenetic analysis of the selected sequences of *orthopoxviruses* (Table 1) by the maximum likelihood method showed that all clades are grouped with high reliability, except for the positions of the branch combining strains CPXV FM2292 and Norway-MAN (CPXV-like2 group [7]), and the TATV branch (Figure 1). Camelpox virus (CMLV) and taterapox virus (TATV) form a common clade with VARV. This allowed the use of TATV and CMLV as the outgroup in the subsequent MCMC analysis of all known sequences of VARV strains.

### 3.2. Molecular Evolution of VARV

**Figure 2:**
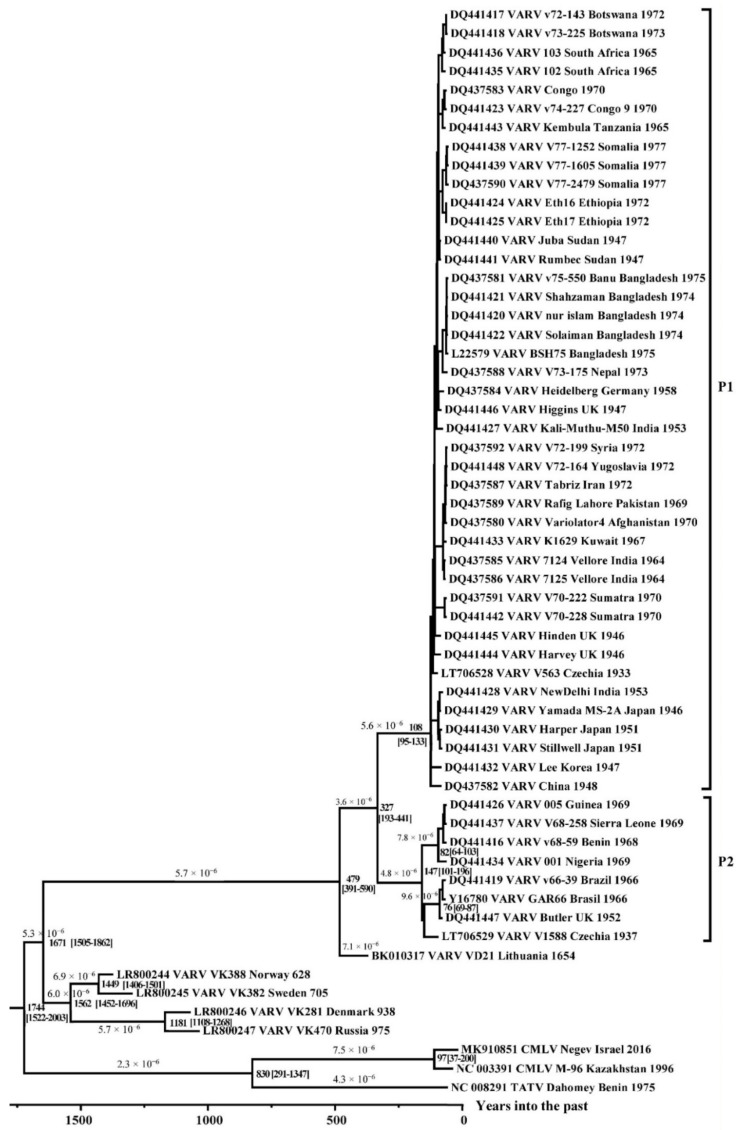
Maximum clade credibility tree for the highly conserved central genome region of VARV, CMLV, and TATV. The chronogram was generated using BEAST 2 software. A log-normal relaxed clock and coalescent Bayesian skyline population prior were used, as well as a HKY substitution model with unequal base frequencies, invariant sites, and gamma-distributed rate heterogeneity among sites. The taxon name fields indicate: the GenBank accession number, the virus, the strain name, the region of sequence origin, and the collection date. The numbers on the nodes indicate the time to the most recent common ancestor (tMRCA) of the clades (years ago) with the 95% highest posterior density (HPD) interval given in square brackets. The rates of mutation accumulation are shown near the branches (substitutions/site/year). The strains are designated as in Table 1.

The chronogram (Figure 2) was constructed using the BEAST 2 program based on the log-normal relaxed molecular clock and coalescent Bayesian skyline population prior. We assessed the degree of the clock-like structure in the data based on a regression of root-to-tip genetic distances against a year of sampling. The obtained significant differences in the rate of evolution of *orthopoxviruses* indicate the need for a relaxed molecular clock. The posterior probability of all nodes, for which the divergence times are given (Figure 1), was 1. The VARV strains VK388, VK382, VK281, and VK470 studied by Mühlemann et al. [2] were isolated in northern Europe and dated back to the 7th–10th centuries AD. In our study, these strains are divided into two groups and form a specific VARV branch, which separated about 1670 (95% highest posterior density [HPD] 1505–1862) years ago from the most recent common ancestor (MRCA) for VARV. The single ancient VARV strain VD21 from Lithuania, dated 17th century AD by Duggan et al. [12], separated about 480 (95% HPD 391–590) years ago from the common MRCA for it and the VARV 20th century strains.

The division of the VARV clades P1 and P2 (Figure 2, Table 2) took place about 327 (95% HPD 193–441) years ago, which corresponds to 1654. Within the P2 VARV group, variola minor alastrim strains from South America and strains from West Africa are divided into two clades (Figure 2). Their divergence occurred about 150 (95% HPD 101–196) years ago, which corresponds to about 1874. On the constructed chronogram (Figure 2), the V1588 strain from the Czech Republic forms a common group with the P2 variola minor alastrim strains from South America and separated from the MRCA about 140 years ago. The time to the most common recent ancestor (tMRCA) of the P1 group VARV strains dates back to the beginning of the 20th century; these strains occurred about 110 (95% HPD 95–133) years ago (Figure 2, Table 2). Strains from the Far East and India from the P1 clade are located closest to the root; they date back to the years 1946–1953; the strains from the Czech Republic (V563, about 1933 AD) and from the United Kingdom (1946 AD) are closest to them. Most of the other strains from the VARV clade P1 were isolated in the 1960s to 1970s.

### 3.3. Molecular Evolution of Orthopoxviruses

**Figure 3:**
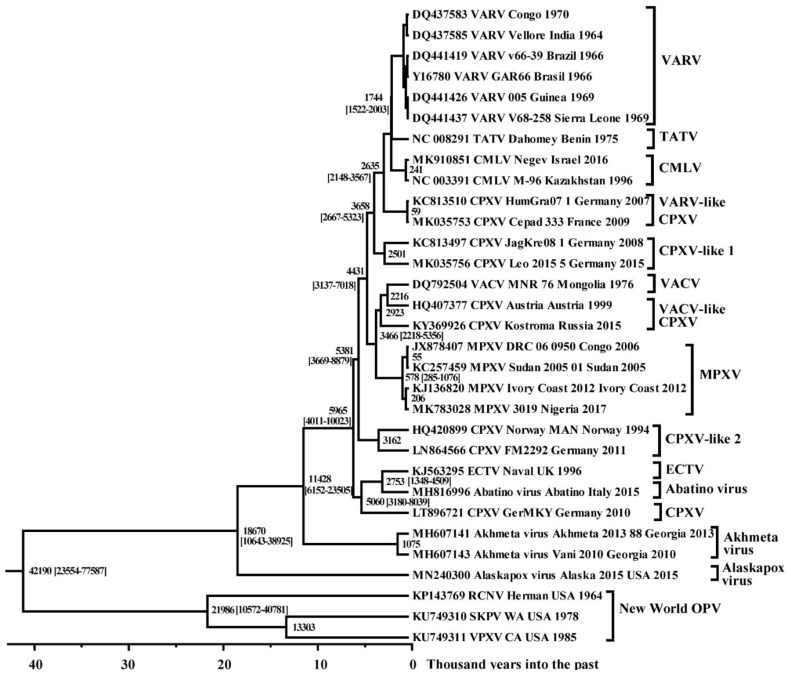
Maximum clade credibility tree for the highly conserved central genome region of the *orthopoxviruses*. The chronogram was generated using BEAST 2 software. A log-normal relaxed clock and coalescent Bayesian skyline population prior were used, as well as a HKY substitution model with unequal base frequencies, invariant sites, and gamma distributed rate heterogeneity among sites. The taxon name fields indicate: the GenBank accession number, the virus, the strain name, the region of sequence origin, and the collection date. The numbers on the nodes indicate the time to the most recent common ancestor (tMRCA) of the clades (years ago) with the 95% highest posterior density (HPD) interval given in square brackets. The strains are designated as in Table 1.

From the analysis of the chronogram (Figure 2), it follows that the division of MRCA into VARV, CMLV, and TATV occurred about 1744 (95% HPD 1522–2003) years ago, which corresponds to about 272 AD. This dating was used to estimate the time of origin of various orthopoxvirus species. A chronogram (Figure 3) was built in the BEAST 2 program using the log-normal relaxed clock and coalescent Bayesian skyline population prior. A previously constructed phylogram (Figure 1) was used as an initial tree. The tree topology has been saved. Notably, the division of *orthopoxviruses* into strains of the Old and New Worlds is estimated to have occurred about 42,000 (95% HPD 24,000–78,000) years ago according to the results of the analysis. The ancestor of raccoonpox virus separated from skunkpox virus and volepox virus of the New World about 22,000 (95% HPD 11,000–41,000) years ago. The last viruses, in turn, split about 13,000 years ago.

It should be noted that Alaskapox virus and Akhmeta virus, belonging to the Old World *orthopoxviruses*, separated approximately 19,000 (95% HPD 11,000–39,000) and 11,000 (95% HPD 6000–24,000) years ago, respectively (Figure 3). The branch combining ECTV, Abatino virus, and CPXV strain GemMKY split off approximately 6000 (4000–10,000) years ago. All other strains of the Old World *orthopoxviruses* began their independent evolution approximately 5000 (95% HPD 4000–9000) years ago; the separation of MPXV took place about 3500 (95% HPD 2200–5400) years ago within this clade. The further appearance of the MPXV West African subtype occurred about 600 (95% HPD 300–1000) years ago.

## 4. Discussion

Despite the eradication of smallpox and further destruction of VARV in the vast majority of collections, a wide variety of other Old World *orthopoxviruses* that are dangerous for humans, such as MPXV, remain in nature. Modern CPXV can be considered the closest relative of the progenitor of VARV, MPXV, VACV, CMLV, TATV, and ECTV (Figure 1) [32,33,34,35,36]. Recently, more evolutionarily ancient *orthopoxviruses* pathogenic for humans have been discovered: Alaskapox virus and Akhmeta virus [9,10]. The discovery of these viruses suggests a natural reservoir of *orthopoxviruses* that are different from other Old World *orthopoxviruses*. In addition, in 2015 Gruber et al. [11] isolated a new Abatino virus pathogenic for a non-human primate. This indicates the insufficient level of our knowledge about the biodiversity of existing *orthopoxviruses*. In this regard, the assessment of the evolutionary potential of *orthopoxviruses* is an urgent task that requires a comprehensive study.

Considering new VARV sequences from ancient burials, we re-calculated the rate of molecular evolution of *orthopoxviruses* based on the highly conserved central genome region of viruses (Figure 2). The genes under conservative selection are mainly located in this area [32,33]. This approach makes it possible to study the evolutionary variability of *orthopoxviruses* with greater accuracy and reliability, because a significant number of the genes localized in the terminal variable regions of the *orthopoxvirus* genomes are under adaptive selection. The probability of recombination events in these regions is high [22,23], a factor that can lead to inaccurate results. We carried out a retrospective analysis using the BEAST 2 program, based on the log-normal relaxed molecular clock and coalescent Bayesian skyline population prior. A log-normal relaxed molecular clock was used due to the inapplicability of а strict molecular clock, which was previously shown using the likelihood ratio test [20] and comparison of the molecular clock [2]. When analyzing VARV strains using CMLV and TATV as an outgroup (Figure 2), the accumulation rate of nucleotide substitutions was 4.4 × 10^−6^ (95% HPD 3.5 × 10^−6^ to 5.3 × 10^−6^) substitutions/site/year. The range of values in different branches varied from 1.6 × 10^−6^ to 8.2 × 10^−6^ substitutions/site/year. These values are consistent with estimates of the rate of variation of the VARV genomes obtained by Mühlemann et al. [2] and Duggan et al. [12].

A group of ancient northern European strains—VK388, VK382, VK281, and VK470 [2]—began their independent evolution from other VARV strains around 350 AD. Mühlemann et al. [2] calculated a slightly later date: 507 AD. We estimated the time of separation of the ancient Lithuania strain VD21 [12] from other VARV strains as 1542 AD, which is in good agreement with the estimates of Mühlemann et al., (1577 AD) [2] and Duggan et al. (1598 AD, using a relaxed molecular clock) [12].

As was first established by Babkina et al. [37], all VARV strains of the 20th century are divided into two clades, namely P1 and P2 (Figure 2). According to our updated data, their separation took place around 1694 AD (Table 2); this is consistent with the dating of Mühlemann et al. [2], Pajer et al. [14], Smithson et al. [15], and Duggan et al. [12]. tMRCA of VARV strains of PI and P2 groups in our work was estimated as 1908 and 1878 AD, respectively (Table 2). These time estimates indicate the recent origin of the biodiversity of the studied VARV strains, and it has been suggested that large-scale vaccination may be the main reason for this [12,38]. In addition, the analysis of a limited set of strains isolated mostly during large outbreaks of smallpox, may not reflect the entire genetic diversity of the VARV strains circulated in the middle of the 20th century. There is little genetic diversity within the P1 group; however, historical records indicate the existence of VARV strains with different pathogenicity [1]. Indeed, analysis of the variability of viral genomes caused by large-scale vaccination can be a very interesting task. The variability of VARV genomes was suggested to be decreased after the large-scale administration of the VACV vaccination. Such reduction of the number of circulating strains was noted for some RNA-viruses after the use of vaccines and therapies [38,39]. However, a small set of available VARV strains circulated before the vaccination program does not allow for such an observation.

VARV strains of the P2 clade cause smallpox, characterized by a significantly lower mortality rate (< 1%) than strains of the P1 clade causing variola major with a mortality rate of 8%–12% [22]. It can be noted that clade P2 contains variola minor alastrim strains from South America, strains from West Africa, and a strain from Europe (Figure 2). However, the issue of the place of origin of these virus variants remains open. It is known that a triangular trading system, carrying slaves, cash crops, and manufactured goods between West Africa, South American colonies, and Europe, had been actively developing from the end of the 16th century [40]. A large number of slaves transported along this route contributed to the spread of VARV strains between the continents. At the same time, extensive smallpox epidemics—especially in South America, where a significant part of the population died due to epidemics after contacts with Europeans [1,41]—contributed to the accelerated evolution of VARV and the emergence of minor alastrim strains.

With the updated data on ancient DNA, it became possible to recalculate the time of VARV emergence. In 2012, Biagini et al. [42] had estimated the time of origin of VARV as far back as 120 AD; however, this estimate was based on a comparative analysis of the short sequence of the ancient PoxSib VARV strain isolated from a burial found in permafrost in Yakutia, Russia. MRCA dating of VARV, CMLV, and TATV based on the genome-wide sequences of ancient VARV strains was not performed. In this study, the time of origin of VARV was first calculated using ancient VARV strains; it was estimated as 272 (95% HPD 13–494) AD.

Analysis of the P1 VARV group shows the trends of grouping these 20th century strains by geography, with VARV strains from India present in most of these groups (Figure 2). This indicates the great biodiversity of VARV strains that circulated in India in the 20th century.

Reconstruction of the evolutionary history of different species of *orthopoxviruses* (Figure 3) allows us to conclude that the *orthopoxviruses* of the Old and New World separated about 42,000 years ago. Based on data on the level of the world ocean in different historical epochs, the existence of an intermittent connection of 60,000–30,000 years ago between North America and Asia in the Beringia region was established [43], which created the possibility of animal migration and the spread of *orthopoxviruses* between continents.

Our retrospective analysis showed that Alaskapox virus separated from its *orthopoxvirus* ancestor about 19,000 years ago, and Akhmeta virus separated about 11,000 years ago (Figure 3). Unfortunately, the natural host of the Alaskapox virus is not known [11]. Akhmeta virus has been identified in both humans and rodents [9,44,45]. Serological testing on the farm where the infected herders worked revealed the presence of anti-*orthopoxvirus* IgG in humans, cows, and wild rodents [45]. This indicates the existence of a natural reservoir of this virus in the territory of Georgia. Then, about 6000 years ago, the MRCA was divided into other modern species of the Old World *orthopoxviruses* (Figure 3). The first to separate was the group combining ECTV, CPXV strain GerMKY, and Abatino virus. These viruses have a different range of susceptible hosts: CPXV has a wide range of hosts, ECTV is pathogenic for rodents, and Abatino virus infects non-human primates. In this group of viruses, CPXV was first isolated about 5000 years ago, then ECTV and the Abatino virus separated about 2800 years ago.

The CPXV-like 2 clade was released about 5400 years ago (Figure 3). Then, about 4400 years ago, a clade was formed, consisting of MPXV, VACV, and VACV-like CPXV. The independent evolution of MPXV began about 3500 years ago. It can be noted that the MPXV West African subtype, which causes a disease with a low mortality rate, appeared relatively recently—about 600 years ago. VACV separated from its closest common ancestor with CPXV about 2200 years ago. A single VACV MNR 76 strain was used in the analysis for the reasons described above.

Approximately 3700 years ago, the clade CPXV-like 1 separated, and then 2600 years ago, the clade VARV-like CPXV segregated (Figure 3). Notably, CPXV does not form one common group based on phylogeny. It can be assumed that CPXV-like viruses were the ancestors of modern Old World *orthopoxviruses*, with the exception of Alaskapox virus and Akhmeta virus. This raises the necessity of revising the CPXV taxonomy.

**Figure 4:**
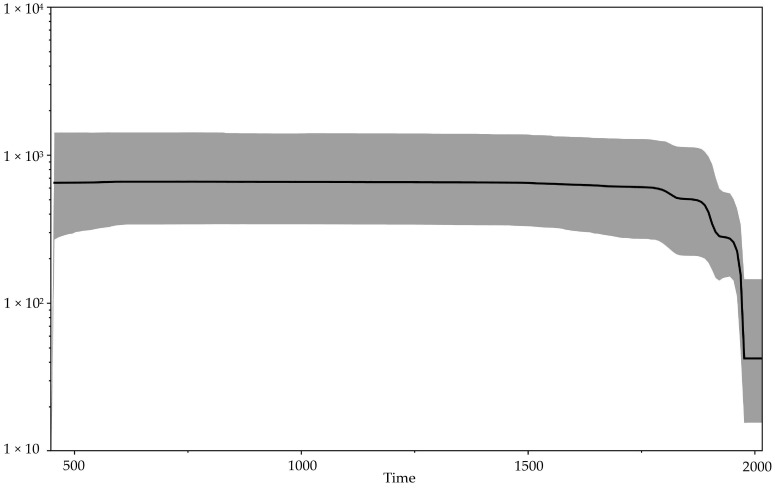
Bayesian skyline plot showing the demographic history of the *orthopoxviruses*. The x axis is in units of years, while the y axis represents effective population size. The black line represents the median value of the population size, and the 95% HPD (highest posterior density) is shown by gray area.

Our updated reconstruction of the evolutionary history of *orthopoxviruses* using modern data on ancient viral VARV DNA indicates a relatively recent emergence of VARV—approximately 272 (95% HPD 13–494) AD (Figure 2, Table 2). This is consistent with the absence of smallpox description in more ancient sources. At the same time, the first reliable description of smallpox referred to the Chinese manuscripts of the 4th century, in which India is named as the source of the disease [1,41]. Some researchers believe that the first descriptions of smallpox are given in the ancient Indian treatises Charaka Samhita and Sushruta Samhita, which were presumably created in the 1st–4th centuries AD [41]. Mühlemann et al. [2] showed that smallpox existed in the early 7th century AD in northern Europe (VARV VK388 from Norway). It appears that in the first centuries of Common Era, the CPXV-like virus with a wide range of hosts became the progenitor of three highly specialized viruses: VARV, CMLV, and TATV. The hosts of the latter two viruses are camels and sole gerbils (*Gerbilliscus kempi*), respectively. Sole gerbils live in the savannas and dry forests of Africa. The overlap of the habitats of these animals indicates the probable origin of these viruses in the Middle East approximately in the 3rd century AD. The significance of such a biogeographic calibration was assessed by Ho et al. [46]. In a relatively short period of time, VARV spread widely across Europe, Africa, and Asia. Mühlemann et al. [2] showed that in the early history of VARV, there were different genovariants of the virus. In the process of evolutionary selection, a single variant of the virus was selected from the pool of ancient VARV strains, which subsequently resulted in the emergence of all modern VARV strains. Vaccination, proposed in 1796 by Edward Jenner and initially used occasionally and locally for restricted groups of people, gradually began to contribute to the evolution of VARV [1]. The Bayesian skyline plot of the demographic population history of VARV, CMLV, and TATV (Figure 4)—showing the change in the levels of population genetic variability, which is equal to the effective population size—reflects this process. A gradual decrease in the effective population size of the above-mentioned viruses can be noted since about 1800. From the beginning of the campaign of smallpox eradication through large-scale vaccination of the population, which was carried out from 1966 to 1977, there has been a sharp drop in the effective population size.

The dating of *orthopoxviruses* in this study is a relative value, based on the smallpox virus, and may be inaccurate. The solution to this problem is possible with further discovery and extensive studies of ancient orthopoxviral DNA. New paleontological data on the expansion of the habitat of the *orthopoxviruses* hosts and consideration of biogeographic calibrations can also improve the dating. Further increasing knowledge will allow the evolutionary history of *orthopoxviruses* to be reconstructed with much greater accuracy.

In conclusion, the emergence of new genetic data has pushed the date of VARV origin from 3000–4000 years ago to about 1700 years ago. This indicates a higher rate of variability and evolutionary potential of pathogenic *orthopoxviruses* than previously thought. Circulation of various genovariants of CPXV and the recently discovered Alaskapox virus and Akhmeta virus in nature can lead to the emergence of new variants of *orthopoxviruses* with unknown properties. Further study of ancient fossil viral pathogens and the search for new *orthopoxviruses* circulating in nature will deepen our understanding of the evolutionary history of these viruses.

## Figures and Tables

**Table 1:** Genome sequences of the *orthopoxviruses* included in the analyses.

No	Species	Strain Name	Country of Isolation	Year of Isolation	Accession Number
1.	Abatino-virus	Abatino	Italy	2015	MH816996
2.	Akhmeta-virus	Akhmeta-88	Georgia	2013	MH607141
3.	Akhmeta-virus	Vani-2010	Georgia	2010	MH607143
4.	Alaskapox-virus	Alaska-2015	The USA **	2015	MN240300
5.	CMLV	M-96	Kazakhstan	1996	NC003391
6.	CMLV	Negev	Israel	2016	MK910851
7.	CPXV	Austria	Austria	1999	HQ407377
8.	CPXV	Cepad-333	France	2009	MK035753
9.	CPXV	FM2292	Germany	2011	LN864566
10.	CPXV	GerMKY	Germany	2010	LT896721
11.	CPXV	HumGra07-1	Germany	2007	KC813510
12.	CPXV	JagKre08-1	Germany	2008	KC813497
13.	CPXV	Kostroma	Russia	2015	KY369926
14.	CPXV	Leo-2015-5	Germany	2015	MK035756
15.	CPXV	Norway-MAN	Norway	1994	HQ420899
16.	ECTV	Naval	The UK	1996	KJ563295
17.	MPXV	3019	Nigeria	2017	MK783028
18.	MPXV	DRC-6-0950	Congo	2006	JX878407
19.	MPXV	IvoryCoast2012	Ivory Coast	2012	KJ136820
20.	MPXV	Sudan-2005-01	Sudan	2005	KC257459
21.	RCNV	Herman	The USA	1964	KP143769
22.	SKPV	WA	The USA	1978	KU749310
23.	TATV	Dahomey	Benin	1975	NC008291
24.	VACV	MNR-76	Mongolia	1976	DQ792504
25.	VARV	001	Nigeria	1969	DQ441434
26.	VARV	005	Guinea	1969	DQ441426
27.	VARV	102	South-Africa	1965	DQ441435
28.	VARV	103	South-Africa	1965	DQ441436
29.	VARV	7124-Vellore	India	1964	DQ437585
30.	VARV	7125-Vellore	India	1964	DQ437586
31.	VARV	BSH75	Bangladesh	1975	L22579
32.	VARV	Butler	The UK *	1952	DQ441447
33.	VARV	China Horn	China	1948	DQ437582
34.	VARV	Congo-9	Congo	1970	DQ437583
35.	VARV	Eth16	Ethiopia	1972	DQ441424
36.	VARV	Eth17	Ethiopia	1972	DQ441425
37.	VARV	GAR66	Brazil	1966	Y16780
38.	VARV	Harper	Japan	1951	DQ441430
39.	VARV	Harvey	The UK	1946	DQ441444
40.	VARV	Heidelberg	Germany	1958	DQ437584
41.	VARV	Higgins	The UK	1947	DQ441446
42.	VARV	Hinden	The UK	1946	DQ441445
43.	VARV	Juba	Sudan	1947	DQ441440
44.	VARV	K1629	Kuwait	1967	DQ441433
45.	VARV	KaliMuthu-M50	India	1953	DQ441427
46.	VARV	Kembula	Tanzania	1965	DQ441443
47.	VARV	Lee	Korea	1947	DQ441432
48.	VARV	NewDelhi	India	1953	DQ441428
49.	VARV	nur-islam	Bangladesh	1974	DQ441420
50.	VARV	Rafig-Lahore	Pakistan	1969	DQ437589
51.	VARV	Rumbec	Sudan	1947	DQ441441
52.	VARV	Shahzaman	Bangladesh	1974	DQ441421
53.	VARV	Solaiman	Bangladesh	1974	DQ441422
54.	VARV	Stillwell	Japan	1951	DQ441431
55.	VARV	Tabriz	Iran	1972	DQ437587
56.	VARV	V1588	Czechia	1937	LT706529
57.	VARV	V563	Czechia	1933	LT706528
58.	VARV	v66-39	Brazil	1966	DQ441419
59.	VARV	V68-258	Sierra-Leone	1969	DQ441437
60.	VARV	v68-59	Benin	1968	DQ441416
61.	VARV	V70-222	Sumatra	1970	DQ437591
62.	VARV	V70-228	Sumatra	1970	DQ441442
63.	VARV	v72-143	Botswana	1972	DQ441417
64.	VARV	V72-164	Yugoslavia	1972	DQ441448
65.	VARV	V72-199	Syria	1972	DQ437592
66.	VARV	V73-175	Nepal	1973	DQ437588
67.	VARV	v73-225	Botswana	1973	DQ441418
68.	VARV	v74-227	Congo	1970	DQ441423
69.	VARV	v75-550-Banu	Bangladesh	1975	DQ437581
70.	VARV	V77-1252	Somalia	1977	DQ441438
71.	VARV	V77-1605	Somalia	1977	DQ441439
72.	VARV	V77-2479	Somalia	1977	DQ437590
73.	VARV	Variolator4	Afghanistan	1970	DQ437580
74.	VARV	VD21	Lithuania	1654	BK010317
75.	VARV	VK281	Denmark	938	LR800246
76.	VARV	VK382	Sweden	705	LR800245
77.	VARV	VK388	Norway	628	LR800244
78.	VARV	VK470	Russia	975	LR800247
79.	VARV	Yamada-MS-2A	Japan	1946	DQ441429
80.	VPXV	CA	The USA	1985	KU749311

Abbreviations: CMLV–camelpox virus; CPXV–cowpox virus; ECTV–ectromelia virus; MPXV–monkeypox virus; RCNV–raccoonpox virus; SKPV–skunkpox virus; TATV–taterapox virus; VARV–variola virus; VPXV–volepox virus. * The strain VARV Butler was imported from South America. ** The strain Alaskapox-virus was probably imported from the Old World region.

**Table 2:** Comparison of the time to the most recent common ancestor (tMRCA) of various *orthopoxvirus* clades with previously published estimates.

	Genome Region and Approach used for Timing					
	Complete VARV Genomes; Strict Clock [12]	Complete VARV Genomes; Relaxed Clock [14]	Complete VARV Genomes; Relaxed Clock [13]	Conservative Region of VARV Genomes; Strict Clock [15]	Complete VARV Genomes; Relaxed Clock [2]	Conservative Region of *Orthopoxvirus* Genomes; Relaxed Clock, this Study
tMRCA–VARV/CMLV/TATV	-	-	-	-	-	272 (13–494)
tMRCA–P1/P2 VARV	1764 (1734–1793)	1695	1809 (1797–1820)	1623 (1579–1667)	1705 (1588–1813)	1694 (1580–1828)
tMRCA–P1 VARV	1910 (1902–1917)	1887	1911 (1908–1915)	1881 (1861–1897)	-	1908 (1888–1926)
tMRCA–P2 VARV	1870 (1855–1885)	1808	1886 (1877–1893)	1794 (1754–1828)	-	1878 (1825–1920)

Numbers in parentheses indicate 95% highest posterior density intervals.

## Data Availability

Not applicable.
